# Using Gut Microbiota Modulation as a Precision Strategy Against Obesity

**DOI:** 10.3390/ijms26136282

**Published:** 2025-06-29

**Authors:** Kwang-Rim Baek, Saloni Singh, Hye-Seon Hwang, Seung-Oh Seo

**Affiliations:** 1 Department of Food Science and Biotechnology, Seoul National University of Science and Technology, Seoul 01811, Republic of Korea; rimmy@seoultech.ac.kr (K.-R.B.); salonisingh@seoultech.ac.kr (S.S.); hhbbgu2@seoultech.ac.kr (H.-S.H.); 2 Research Institute of Food and Biotechnology, Seoul National University of Science and Technology, Seoul 01811, Republic of Korea

**Keywords:** gut microbiota, obesity, metabolic disorder, host–microbe interaction, microbiome engineering

## Abstract

Obesity is a complex metabolic disorder with high global prevalence. Recent studies have highlighted the crucial role of gut microbiota in obesity’s onset and progression. This review explores the relationship between gut microbiota composition and obesity, emphasizing how changes in microbial communities can influence host metabolism, energy balance, and fat storage. By reviewing current evidence regarding the interactions between specific microbial taxa; their metabolic byproducts, such as short-chain fatty acids; and host signaling pathways, we aim to clarify the mechanisms through which the gut microbiome contributes to obesity. Furthermore, we discuss the potential of microbiota engineering through precision strategies such as the use of probiotics, prebiotics, and genetically engineered microbial strains. Collectively, this review highlights the targeted modulation of the gut microbiome as a promising and innovative approach to the prevention and treatment of obesity.

## 1. Introduction

Obesity represents a significant global health challenge, characterized by excessive adipose tissue accumulation, and is associated with an increased risk of metabolic disorders, including type 2 diabetes, cardiovascular disease, and certain malignancies [1]. It is a multifaceted medical condition, typically defined as a body mass index (BMI) of 30 or higher, resulting from an imbalance between caloric intake and energy expenditure [2]. Obesity is not merely a result of consuming too many calories; it is a complex disorder influenced by genetic, metabolic, environmental, and behavioral factors. Diverse determinants shape this multifaceted condition. Achieving long-term weight loss is often challenging because of physiological changes, such as a reduced basal metabolic rate and increased hunger during calorie restriction. While traditional strategies, including dietary interventions such as low-fat, low-carbohydrate Mediterranean diets and intermittent fasting, can facilitate weight loss through an energy deficit, their success largely hinges on consistent adherence. Pharmacological treatments, such as GLP-1 receptor agonists (e.g., liraglutide and semaglutide), have demonstrated effectiveness in enhancing satiety and glycemic control. However, they often require long-term use and may involve side effects or varying individual responses [3].

Despite these limitations, the gut microbiota has emerged as a crucial and potentially transformative element in the management of obesity. In addition to its traditional role in digestion, the gut microbial community significantly influences host metabolism, energy homeostasis, fat storage, and appetite regulation. Often conceptualized as a “virtual organ,” the gut microbiome plays essential roles in host metabolism, energy balance, and fat storage. Over the past decade, our understanding of the involvement of the gut microbiota in obesity has greatly expanded, revealing a complex and dynamic interplay between microbial communities and host metabolism. Rather than being passive entities, gut microbes actively participate in energy regulation and fat storage. For instance, individuals with obesity often exhibit a higher proportion of Firmicutes than Bacteroidetes, a shift that enhances the conversion of otherwise indigestible dietary fibers into absorbable short-chain fatty acids (SCFAs), thereby extracting more calories from the same amount of food [4]. These SCFAs, particularly acetate, propionate, and butyrate, are not merely byproducts but also serve as essential metabolic signals. By activating receptors such as G-protein-coupled receptor (GPR)41 and GPR43, they influence energy expenditure and fat accumulation and even modulate the release of satiety hormones such as GLP-1 and PYY, which help regulate appetite and improve insulin sensitivity [5,6].

The gut microbiota is essential for maintaining the integrity of the intestinal barrier, extending beyond its role in metabolism. In instances of dysbiosis, characterized by an imbalance in the microbial community, this barrier may become compromised, allowing bacterial endotoxins, such as lipopolysaccharides (LPSs), to enter the bloodstream. This process initiates a state of chronic low-grade inflammation that is directly associated with insulin resistance and obesity [7]. Moreover, the gut microbiota modulates bile acid metabolism, which subsequently influences nuclear receptors such as Farnesoid X Receptor (FXR) and Takeda G-protein-coupled Receptor 5 (TGR5), thereby further affecting lipid and glucose homeostasis [8]. These findings collectively highlight the gut microbiome as not just a reflection of metabolic health but also as a modifiable determinant. Consequently, strategies focused on engineering or reshaping gut microbiota through probiotics, prebiotics, or synthetic microbial therapies hold significant promise for addressing obesity.

This review aims to investigate the relationship between gut microbiota composition and obesity, with a specific focus on how gut microbes influence weight gain and metabolic health. Moreover, we examine the potential of microbiome engineering, including the use of probiotics, prebiotics, and genetically modified microbial strains, as a promising and innovative approach to the prevention and treatment of obesity.

## 2. The Relationship Between the Gut Microbiome and Obesity

### 2.1. Dysbiosis of Gut Microbiome

**Figure 1:**
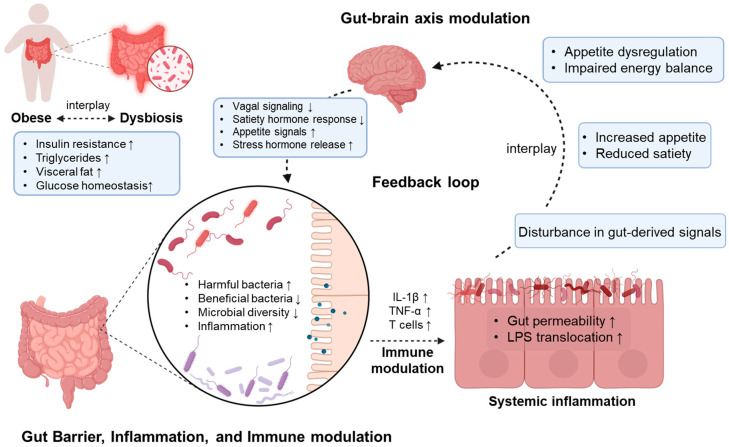
Relationship between obesity and gut microbiome.

The gut microbiome can interact with the host to regulate physiological and metabolic functions such as energy balance and hormonal changes [4,9]. The gut microbiome shares nutrients with the host as a part of a symbiotic relationship [10,11,12]. For example, non-digestible sugars can be fed to gut microorganisms that ferment them into monosaccharides and short-chain fatty acids, which can then be used as nutrients by the host [4,13]. The metabolites of the gut microbiome, such as SCFAs, LPSs, and other components (e.g., peptidoglycans, extracellular vesicles), can also be involved in host metabolism by affecting the gut, adipose tissue, brain, and other organs [9,10,14]. Although the mechanisms underlying host–microbiome interactions are not yet fully understood, emerging evidence suggests their involvement in various diseases [15]. The gut microbiome is increasingly recognized as having endocrine-like functions that regulate energy homeostasis and fat storage [16,17]. Therefore, dysbiosis is considered to be an important factor in obesity [16] (Figure 1).

The gut microbiome can be broadly divided into five phyla, Bacteroidetes, Firmicutes, Actinobacteria, Proteobacteria, and Verrucomicrobia, among which Bacteroidetes and Firmicutes are the most abundant [18,19,20]. Studies have reported that changes in the ratio of Firmicutes to Bacteroidetes (F/B ratio) are associated with obesity [21,22]. Andoh et al. (2016) analyzed the gut microbiome of obese (BMI > 35) and lean (BMI < 20) individuals in Japan using fecal samples [21]. Their study found that Firmicutes were significantly more abundant in obese individuals, while lean individuals showed increased levels of anti-inflammatory bacteria, such as *Faecalibacterium prausnitzii*. Pro-inflammatory species, such as *Bacteroides vulgatus*, are increased in obese individuals [21]. Chavez-Carbajal et al. (2019) compared the gut microbiome of healthy (CO), obese (OB), and obese–metabolic syndrome (OMS) Mexican women [22]. The OB (72.97%) and OMS (73.34%) groups had a higher Firmicutes percentage than the CO group (56.9%), with beta diversity analysis showing a separation between the CO, OB, and OMS groups [22]. Although the F/B ratio has been proposed as a marker of obesity-associated dysbiosis, subsequent studies have shown inconsistent results due to differences in diet, host genetics, and environmental factors [13,23]. Borgo et al. (2018) found that the F/B ratio was not associated with obesity [24]. Their study of normal (BMI < 25) and obese (BMI > 30) subjects analyzed both luminal (LAM) and mucosal (MAM) microbiomes. While LAM samples showed reduced α-diversity in obese individuals compared to that in normal individuals, the F/B ratio did not differ significantly between the two groups. Notably, two key anti-inflammatory taxa, *Faecalibacterium prausnitzii* and *Flavonifractor plautii*, were depleted in obese microbiota, suggesting a link between obesity and the loss of beneficial microbes [24].

In line with this, recent studies have focused on reducing the gut microbiome’s diversity and the abundance of certain bacteria in obese individuals. Muheyati et al. (2024) investigated obesity and gut microbiota in 47 adults in Urumqi [25]. The results showed that the obese group had reduced microbial diversity compared with the normal-weight group. The relative abundance of short-chain fatty acid-producing bacteria, including Ruminococcaceae and Coprococcus, was lower in the obese group. The obese group also had elevated serum leptin and C-reactive protein (CRP) levels, indicating higher systemic inflammation. These findings suggest that specific gut microbiota may influence inflammation and metabolic pathways in obesity [25]. Baoting et al. (2025) investigated the association between the gut microbiome and obesity in adolescents using the Children of 1997 birth cohort [26]. An analysis of fecal samples showed that obese adolescents had significantly lower microbial richness and diversity, although the F/B ratio did not differ significantly between groups. At the species level, the relative abundance of *Bacteroides uniformis*, a species associated with a reduced obesity risk, was significantly lower in the obese group than in the control group. Metabolomics also revealed an inverse association between *B. uniformis* and branched-chain amino acids (BCAAs), indicating their potential relevance not only to obesity but also to type 2 diabetes [26]. Another study examined the gut microbiota in 114 Colombian adults [27] and showed that lower Clostridia diversity was associated with a higher prevalence of obesity. These studies have highlighted the role of gut microbial diversity in obesity and overall metabolic health [27].

### 2.2. SCFA Production by Gut Microbiome

Short-chain fatty acids (SCFAs) are fermentation products of microorganisms [28]. The gut microbiota can break down sugars that cannot be degraded by human digestive enzymes such as oligosaccharides, inulin, and resistant starch into smaller molecules through fermentation [11,29]. The SCFAs produced during fermentation are used as an energy source for intestinal cells and transported to peripheral tissues for further metabolism [30,31]. SCFAs are mainly composed of butyrate, propionate, and acetate. [28]. Butyrate is the preferred nutrient for the metabolism and growth of colonic epithelial cells [32]. Propionate is transported by the colon cells to the liver. Once in the liver, propionate serves as a substrate for gluconeogenesis and regulates cholesterol synthesis [33,34]. Acetate serves as an important substrate in the liver and peripheral nerves and is used for lipogenesis and cholesterol synthesis [9,35].

A study by S. O. Johnson et al. (2023) examined the association between obesity, gut microbiota diversity, and SCFA concentrations using data from the METS-microbiome study, which involved a multinational cohort of individuals of African descent [36]. The researchers found that obese participants had reduced microbial diversity and lower fecal SCFA levels, particularly those of butyrate and propionate. These findings suggest that the gut microbiome and its metabolites such as SCFAs are closely linked to obesity [36]. Sanna et al. (2019) analyzed the relationship between SCFAs and metabolic diseases such as obesity and type 2 diabetes using Mendelian randomization (MR) analyses [37]. The results showed that the effects of SCFAs vary depending on their type. For example, an increased abundance of butyrate-producing bacteria, such as *Eubacterium rectale* and *Roseburia intestinalis*, is associated with an increased insulin response. In contrast, higher fecal propionate concentrations are positively associated with BMI and an increased risk of type 2 diabetes. These findings indicate that the health impact of SCFAs is not simply determined by their total concentration but rather by the functional balance and context in which they function [37]. On the other hand, other studies have suggested that excessive SCFAs play a role in hormonal changes and increased inflammation, which may be an important cause of obesity [9,35]. When gut microbiomes from obese and lean mice were transplanted into germ-free (GF) mice, the mice that received the obese microbiome produced more acetate and butyrate. In addition, more SCFAs have been found in the feces of obese individuals than in lean individuals [4,38]. Cuesta-Zuluaga et al. (2018) conducted an epidemiological study on the relationship between SCFAs and metabolic diseases, including obesity, in Colombian populations [39]. The associations between fecal SCFAs, gut microbiota diversity, intestinal permeability, and obesity were examined in 441 adults. Gut microbial diversity and fecal SCFA concentration were inversely correlated, and SCFA concentration was proportionally related to intestinal permeability, a marker of obesity. Consequently, higher SCFA excretion is associated with gut dysbiosis, intestinal permeability, excessive obesity, and cardiometabolic risk factors [39]. These findings suggest that fecal SCFAs may play opposing roles in obesity, depending on the context. While fecal SCFAs are commonly used as indicators of gut microbial fermentation, they may not accurately represent the physiological activity of SCFAs given that approximately 95% of colonic SCFAs are absorbed and only a small amount is excreted in feces [33,40]. Müller et al. (2019) investigated the relationship between fecal and circulating SCFA concentrations and metabolic health markers in a cohort of 160 participants with a wide range of BMI and glucometabolic statuses [40]. Their study found that fecal SCFAs were not significantly associated with insulin sensitivity, blood glucose, or lipolysis, whereas circulating SCFAs were positively associated with improved lipid metabolism and insulin sensitivity [40]. These results highlight the need for further studies to elucidate their underlying mechanisms of action.

### 2.3. Hormonal Modulation by Gut Microbiome

The gut microbiome regulates hormones and the nervous system, influencing insulin secretion, food intake, satiety, and appetite through gut peptides and neurotransmitters [10,41,42]. The gut–brain axis is the bidirectional network that connects the gut and the central nervous system through hormones. Satiety hormones, like GLP-1 and PYY, signal the brain via vagal pathways to reduce appetite and increase satiety. The relationship between hormonal changes in the gut microbiome and obesity can be divided into three mechanisms. First, the gut microbiome directly releases compounds that act like hormones that influence obesity development. Second, metabolites produced by the gut microbiome affect obesity by modulating gut peptides and hormones. Third, neurotransmitters produced by the gut microbiome can affect obesity.

Several studies have identified gut microbes with anti-obesity properties that secrete compounds that act like hormones. For example, GLP-1 is a hormone secreted by enteroendocrine cells following food intake that stimulates insulin secretion. P9, a protein secreted by *Akkermansia muciniphila*, enhances thermogenesis in brown adipose tissue, reduces body weight, and promotes the secretion of GLP-1, thereby contributing to obesity improvement [43]. *Bacteroides acidifaciens* has been reported to activate proliferator-activated receptor α (PPARα) and inhibit dipeptidyl peptidase-4 (DPP-4) secretion, resulting in anti-obesity effects [44]. The activation of PPARα promotes fatty acid β-oxidation, leading to weight loss, whereas DPP-4 is an enzyme that degrades GLP-1, thereby modulating satiety signals [44].

Metabolites, such as SCFAs, produced by the gut microbiome may regulate obesity by affecting the central nervous system or modulating the secretion of gut hormones [45]. SCFAs trigger the secretion of gut peptides, such as GLP-1 and PYY, by binding to G-protein-coupled receptors (GPCRs), such as FFAR2/GPR43 and FFAR3/GPR41, expressed in gut epithelial cells. Studies have shown that butyrate, propionate, and acetate exert protective effects against diet-induced obesity and insulin resistance. In mouse models fed a high-fat diet (HFD), SCFAs suppressed weight gain and increased the expression of GPR43 and GPR41 in the adipose tissue. In humans, SCFA supplementation in overweight individuals has been shown to enhance energy expenditure and PYY secretion, thereby promoting fat oxidation [46,47,48]. The gut microbiome affects bile acid metabolism by synthesizing intestinal bile acids and converting them into secondary bile acids. These secondary bile acids bind to TGR5, stimulate GLP-1 and insulin secretion, reduce food intake, and improve glucose tolerance [46,48,49].

The gut microbiome produces and modulates the production of neurotransmitters, such as γ-aminobutyric acid (GABA) and serotonin, which are involved in central appetite regulation [50]. GABA, synthesized predominantly by *Levilactobacillus brevis* and *Bifidobacterium dentium*, functions as the principal inhibitory neurotransmitter in the central nervous system and is involved in lipid metabolism and appetite control [51]. The metabolites of gut microbiota, such as SCFAs and secondary bile acids, have been shown to stimulate enterochromaffin (EC) cells, thereby promoting the production of serotonin [52,53].

### 2.4. Increased Lipopolysaccharide (LPS) Levels

Lipopolysaccharide (LPS) is a component of Gram-negative bacterial cell walls that consists of lipids and polysaccharides [54]. Low concentrations of LPS in the human gut help maintain intestinal barrier integrity, whereas higher concentrations can impair this barrier and facilitate LPS translocation into the bloodstream [55]. Basal LPS from commensal bacteria weakly activates TLR4 and supports gut barrier function and motility via GLP-1 release. In contrast, pathogenic LPS strongly activates TLR4, triggering pro-inflammatory cytokines and systemic inflammation [56,57]. This dysfunction is the leading cause of the chronic inflammation associated with obesity [12]. LPS levels increase with a high-fat diet, potentially indicating endotoxemia and contributing to elevated tissue inflammation, which may lead to obesity or obesity-related metabolic diseases [7]. Endogenous LPS produced by Gram-negative bacteria is continuously generated in the gut and absorbed by intestinal capillaries via Toll-like receptor 4 (TLR4) [54,58]. Rats injected with LPS for four weeks showed similar fasting glycemia, insulinemia, and adipocyte growth as rats fed a high-fat diet, suggesting an LPS–obesity association [7]. Additionally, obesity is correlated with an increased abundance of *Firmicutes* bacteria and decreased levels of *Bifidobacterium*. These changes in the proportion of bacteria are associated with elevated plasma LPS levels in mice [7,55]. Cani et al. (2007) showed that LPS is an early factor in HFD-induced metabolic diseases [7]. Mice fed a high-fat carbohydrate-free diet for four weeks showed increased plasma LPS levels, inducing insulin resistance [7]. La Serre et al. (2010) studied gut inflammation and microbiome changes from high-fat diets [59]. Rats fed a high-fat diet for 12 weeks showed increased plasma LPS levels, likely due to increased intestinal permeability, leading to microbial factor leakage [59]. Another study found that a high-fat diet altered gut microbiota and increased LPS levels, inducing metabolic endotoxemia [60]. Mice fed a high-fat diet showed increased intestinal permeability and LPS translocation, leading to obesity, insulin resistance, and adipose tissue inflammation. To confirm that these effects were mediated by gut microbiota, antibiotics were administered along with the high-fat diet. This intervention normalized LPS levels and led to improvements in body weight, insulin sensitivity, inflammation, and fat accumulation [60].

## 3. Current Anti-Obesity Strategies Used by Modulating Gut Microbiome

### 3.1. Dietary Control

**Figure 2:**
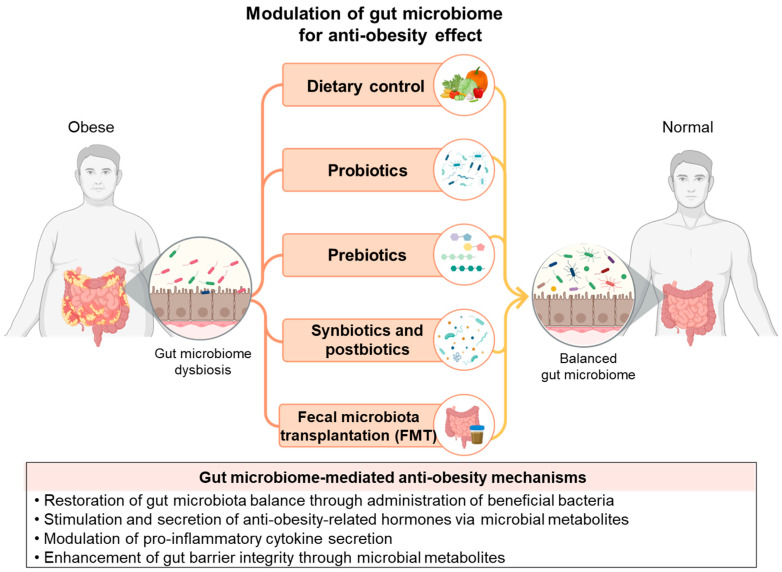
Modulation of gut microbiome for anti-obesity effect.

Improving the gut environment, including the balance of the gut microbiome, has been reported to have beneficial effects on obesity, leading to the development of various therapeutic strategies (Figure 2). Several environmental and lifestyle factors such as diet, exercise, stress, and medication can influence the composition and function of the gut microbiota. Diet plays a central role in shaping the gut microbiome, and approaches such as dietary modification and the use of probiotics, prebiotics, postbiotics, and synbiotics have been proposed as effective means of modulating microbial composition. Fecal microbiota transplantation (FMT) from healthy donors has emerged as a promising strategy for reconstituting the gut microbiome. Accordingly, these strategies have led to the proposal of microbiome-based approaches to obesity treatment (Table 1). Diet-induced microbial changes affect energy absorption, hormone levels, and SCFA production, all of which contribute to obesity [61,62,63,64]. Studies have shown that high-fat diets can disrupt gut microbiota composition, reduce the expression of tight junction proteins, and increase intestinal permeability, leading to LPS leakage, systemic inflammation, and subsequent metabolic complications [65,66]. According to Baothman et al. (2016), a high-fat diet alters the composition of the gut microbiome, leading to systemic inflammation [28]. Do et al. (2018) reported that a high-sugar diet increases lipid accumulation, induces hepatic steatosis, and reduces gut microbial diversity [67]. Western diets with high fat and sugar levels reduce the gut’s microbial diversity and increase the F/B ratio, leading to obesity. In contrast, Mediterranean and vegetarian diets are known for their anti-obesity effects, which increase *Bacteroidetes* and decrease *Proteobacteria* [68].

Dietary fiber is frequently cited as an anti-obesity dietary component. It serves as a substrate for the gut microbiome, stimulates the production of SCFAs, and enhances microbial diversity within the gut [95,96]. Dietary fiber intake is associated with an increase in the SCFA-producing gut microbiota, reduction in the F/B ratio, and improvement in gut microbiome dysbiosis [97]. This anti-obesity effect was confirmed in a comparative study of African and European children, in which African children with higher dietary fiber consumption exhibited a lower F/B ratio, elevated SCFA levels, and greater gut microbiome diversity [98]. Dietary fiber comprises both soluble and insoluble components, each contributing to its anti-obesity effects. Soluble dietary fibers ameliorate obesity by modulating the gut microbiome. In high-fat diet (HFD)-induced obese mice, soluble dietary fiber was found to reduce the F/B ratio and increase the levels of energy-expending microbes, thereby mitigating weight gain and white adipose tissue accumulation [69]. Insoluble dietary fibers derived from sources such as brown seaweed, soybeans, and pear fruit pomace have been shown to reduce the F/B ratio and Lachnospiraceae levels while enhancing the levels of SCFA-producing microbes, leading to decreased body weight, fat mass, and cholesterol levels [70,71,72,73,99]. Notably, bamboo shoot fibers demonstrated superior anti-obesity effects compared to other fibers. They effectively inhibited HFD-induced weight gain in mice and improved glycemic control. A bamboo shoot fiber diet increased the abundance of Bacteroidetes, which was negatively correlated with weight gain, and suppressed Verrucomicrobia, which was positively correlated with weight gain [74].

Several studies have demonstrated the anti-obesity effects of various dietary ingredients through their interaction with the gut microbiome. Berberine (BBR), a Chinese herb, prevents obesity by modulating the gut microbiome and serum brain–gut hormones [75]. In mice with high-fat diet (HFD)-induced obesity, BBR administration increased GLP-1 receptor (GLP-1R) and orexin A expression while decreasing brain neuropeptide Y (NPY) expression, leading to reductions in body weight, plasma lipid levels, and insulin resistance. In addition, BBR administration altered the composition of gut microbiota by decreasing the F/B ratio and increasing the abundance of SCFA-producing bacteria. [75]. Mung bean supplementation exerts anti-obesity effects by modulating gut microbiome dysbiosis. In HFD-induced obese mice, mung beans reduced body weight and fat accumulation and improved glucose tolerance while also modulating the gut microbiota by decreasing the F/B ratio and increasing the levels of beneficial genera such as *Akkermansia* and *Bifidobacterium* [76]. *Hirsutella sinensis* mycelium (HSM) exerts an anti-obesity effect by promoting gut microbiome growth. In HFD-induced obese mice, HSM reduced obesity and insulin resistance. Its polysaccharide fraction reduces body weight by 50% and promotes the growth of *Parabacteroides goldsteinii* [77].

### 3.2. Probiotics

Probiotic supplementation results in both statistically and clinically significant reductions in body weight (BW), body mass index (BMI), and waist circumference (WC) [100,101]. Probiotics influence weight management through various mechanisms, including the restoration of gut microbiota balance, the modulation of energy homeostasis, and lipid metabolism [102]. Probiotic strains contribute to reduced fat storage and inflammation by altering microbial diversity, which is often disrupted in individuals with obesity. At the molecular level, probiotics attenuate hypothalamic inflammation, improve insulin sensitivity, and affect the neuroendocrine regulation of appetite and satiety via vagus nerve signaling. They elevate the levels of key gut peptides such as GLP-1, leptin, cholecystokinin, and peptide YY, all of which enhance satiety and delay gastric emptying. In an animal study, mice administered *Bacteroides acidifaciens* showed a significant reduction in body weight compared with controls, despite having similar food intake [44]. Kim et al. (2025) evaluated the anti-obesity effects of *Lactobacillus paragasseri* SBT2055 (LG2055) in an HFD-induced obese mouse model [78]. The results showed that the administration of LG2055 significantly reduced body weight gain and white adipose tissue accumulation compared to the HFD group. In addition, LG2055 suppressed the expression of genes involved in intestinal fat absorption, including CD36, FATP4, Fabp1/2, and ApoB48. Furthermore, LG2055 treatment was found to increase the diversity of the gut microbiota [78]. Brain et al. (2023) evaluated the therapeutic potential of soy yogurt fermented with GABA-producing *Lactiplantibacillus plantarum* GA30 in a streptozotocin-induced diabetic mouse model [80]. Compared to the soymilk control, GA30 yogurt significantly increased plasma GABA levels (5-fold), reduced hyperglycemia, and attenuated body weight loss [80]. *Lactobacillus gasseri* BNR17 has been shown to reduce obesity by lowering serum leptin levels. In obese mice fed a high-sucrose diet, *L. gasseri* BNR17 suppressed body weight gain and leptin levels [79]. A clinical study involving the probiotic strain *Hafnia alvei* HA4597^®^ showed weight loss in overweight individuals over 12 weeks [82]. The probiotic produces a ClpB protein that mimics satiety, similarly to α-MSH, potentially aiding in appetite regulation [82]. Probiotics also target several inflammatory and metabolic signaling pathways, including Toll-like receptors (TLR2 and TLR4) and the NLRP3 inflammasome, to reduce adipogenesis and promote metabolic health. Furthermore, they influence gut fermentation capacity, improve SCFA production, and enhance gut barrier integrity. These mechanisms are associated with improved lipid profiles, decreased visceral fat accumulation, and reduced systemic inflammation (e.g., lower CRP, IL-6, and TNF-α levels) [101]. In a study by Wu et al. (2025), the anti-obesity effects of *Lacticaseibacillus paracasei* K56 in an HFD-induced obese mouse model were explored, and the underlying mechanisms were analyzed [81]. The administration of *L. paracasei* K56 suppressed HFD-induced body weight gain and epididymal adipose tissue accumulation while also improving blood glucose levels and insulin resistance. In addition, the treatment attenuated inflammatory responses by inhibiting the TLR4/NF-κB and JNK signaling pathways in the colon [81].

Obesity has been linked to reduced levels of SCFA-producing bacteria and an imbalance in the F/B ratio, favoring energy harvesting and fat accumulation. Probiotics help normalize the F/B ratio; promote SCFA production; detoxify bacterial endotoxins, such as LPS; and stimulate the expression of intestinal alkaline phosphatase, thereby reducing metabolic endotoxemia [103]. In addition, dietary components can also disrupt gut balance and contribute to obesity. Advanced glycation end products (AGEs) are abundant in processed foods, particularly those cooked at high temperatures such as fried foods. High-AGE diets have been shown to promote pro-inflammatory bacteria and reduce the levels of beneficial microbes and contribute to the development of obesity and insulin resistance [104]. Unabsorbed AGEs reach the colon, where they interact with the gut microbiota. A recent study has demonstrated that *Cloacibacillus evryensis* can degrade simple AGEs like N(ε)-carboxymethyllysine (CML) into byproducts, thereby facilitating their excretion through feces [105].

Probiotics also regulate the expression of fasting-induced adipose factor, which inhibits lipoprotein lipase activity and reduces fat storage. By enhancing the gut barrier and modulating immune and metabolic signaling, probiotics affect host energy metabolism, reduce appetite, and influence the central and peripheral mechanisms of food intake [106,107].

Probiotics are a promising adjunctive approach in the management of obesity, primarily because of their ability to modulate the gut microbiome. Nevertheless, their efficacy is contingent upon factors such as dosage, duration, strain diversity, and host-specific variables including sex and metabolic status. Optimizing these parameters may result in more consistent and clinically significant outcomes for the prevention and treatment of obesity.

### 3.3. Prebiotics

Prebiotics are non-digestible food components that undergo selective fermentation, thereby benefiting the host by promoting the proliferation and activity of advantageous gut bacteria, notably *Bifidobacterium* and *Lactobacillus* [108]. Common prebiotics include inulin, fructooligosaccharides (FOSs), galactooligosaccharides (GOSs), human milk oligosaccharides (HMOs), and resistant starches, which are naturally present in foods, such as garlic, onions, chicory root, bananas, and whole grains. In contrast to probiotics, which introduce beneficial microorganisms into the gut, prebiotics serve as substrates that nourish and enhance the existing microbiota of the host, thereby fostering the growth of health-promoting bacteria already present in the colon [109]. In a study by Singh et al. (2018), the anti-obesity effects of inulin were investigated in a high-fat diet-induced obese male rat model [83]. The results showed that inulin supplementation reduced caloric intake, body weight gain, and fat accumulation in a dose-dependent manner. In addition, inulin improved blood glucose levels and glucose tolerance and increased the mRNA expression of gut-derived satiety hormones, including CCK, PYY, and GLP-1. Furthermore, inulin modulated gut microbiota composition by increasing the abundance of Bacteroidetes and *Bifidobacterium* spp. while decreasing the levels of *Clostridium* clusters I, IV, and XIV and *Roseburia* [83]. A study by Paone et al. (2022) found that 6 weeks of FOS administration inhibited weight gain and fat accumulation, improved glucose tolerance, and increased gut microbiota, including beneficial bacteria such as *Akkermansia*, *Odoribacter*, *Roseburia*, and *Muribaculaceae*, which are associated with gut barrier function [85]. In a clinical study conducted by Mistry et al. (2020), the effects of GOS supplementation on metabolic health were examined [84]. The results showed that participants who consumed GOS experienced enhancements in their lipid profiles and a decrease in inflammation markers, suggesting possible advantages for metabolic regulation [84]. HMO is known as a natural prebiotic that modulates the gut microbiota. The intake of infant formula supplemented with 2′-fucosyllactose, a key component of HMOs, has been associated with reduced inflammation and a lower incidence of diarrheal disease in infants. Moreover, breastfeeding, which provides HMOs, has been reported to contribute to the prevention of childhood obesity [110,111].

Prebiotics play a multifaceted role in addressing obesity by influencing the composition and metabolic activity of gut microbiota. Their fermentation in the colon enhances the production of SCFAs such as acetate, propionate, and butyrate. These SCFAs regulate host metabolism by binding to G-protein-coupled receptors (GPR41 and GPR43), which are involved in the release of appetite-suppressing hormones, such as glucagon-like peptide-1 (GLP-1) and peptide YY (PYY). These hormones promote satiety, reduce food intake, and improve insulin sensitivity [108,109,112]. Moreover, prebiotics contribute to maintaining gut barrier integrity by increasing mucosal thickness and the expression of tight junction proteins, thereby reducing the endotoxemia caused by lipopolysaccharides (LPSs) from Gram-negative bacteria. Lower systemic LPS levels are associated with reduced chronic inflammation and a decreased risk of insulin resistance, which are both critical factors in bloating and diarrhea, particularly at elevated doses. Furthermore, the optimal dosing and long-term safety have not yet been fully established. Additional large-scale studies are necessary to support standardized clinical applications [113].

### 3.4. Synbiotics and Postbiotics

Synbiotics are combinations of probiotics and prebiotics that were first introduced by Gibson and Roberfroid in 1995 to describe their synergistic effects [114]. Synbiotics may be more effective than probiotics or prebiotics alone because of their synergistic enhancement in probiotic viability in the gut [115,116,117]. Given these synergistic properties, synbiotics have also been studied for their roles in obesity. Several studies have demonstrated the anti-obesity effects of synbiotics. Safavi et al. (2013) observed an anti-obesity effect in children and adolescents aged 6–18 years with BMI above the 85th percentile [86]. The participants were randomly assigned to two groups to receive synbiotics, containing a combination of *Lactobacillus casei*, *L*. *rhamnosus*, *L*. *acidophilus*, *L*. *bulgaricus*, *Streptococcus thermophilus*, *Bifidobacterium breve*, and *B. longum*, with FOS, or placebo for 8 weeks. The synbiotic group exhibited lower BMI, serum triglyceride levels, and total and LDL cholesterol levels than the placebo group [86]. Hadi et al. (2020) conducted a systematic review and meta-analysis of clinical trials to evaluate the effects of synbiotic supplementation on obesity-related outcomes [118]. The results showed that synbiotic supplementation led to statistically significant reductions in body weight and waist circumference. These effects are likely mediated by the modulation of gut microbiota composition, suggesting a potential role for synbiotics in the treatment of obesity [118]. Khanna et al. (2021) utilized *L. pentosus* GSSK2 with isomalto-oligosaccharides to investigate their effects in high-fat diet (HFD)-induced obese mice [87]. Mice were divided into four groups: HFD, HFD + prebiotics, HFD + probiotics, and HFD + synbiotics. Among these, the synbiotic group showed the most significant anti-obesity effects, including reduced weight gain and adipose tissue accumulation as well as improved glucose tolerance. Furthermore, synbiotic treatment improves gut microbiota dysbiosis by shifting the composition from obesity-associated bacteria to non-obesity-associated bacteria [87]. Thus, synbiotics have been found to be more effective in preventing obesity-induced gut microbiome dysbiosis than either component of them alone, as prebiotics facilitate probiotic growth [119].

According to the International Scientific Association for Probiotics and Prebiotics (ISAPP), the “preparation of inanimate microorganisms and/or their components confer a health benefit on the host”, as shown in postbiotics [120]. This concept is based on the metabolites, fractions, and cell lysates of microorganisms that provide bioactivity. There are two main types of microbial metabolites and their components. Microbial metabolites include enzymes, proteins/peptides, exopolysaccharides, organic acids, vitamins, phenolic-derived metabolites, aromatic amino acids, and lipids such as SCFAs. Microbial components include cell wall components such as lipoteichoic acids, teichoic acids, peptidoglycan, cell surface proteins, and polysaccharides [121,122]. Compared with probiotics, postbiotics are associated with a reduced risk of infection, bacterial translocation, and antibiotic resistance gene transfer and are easier to transport and store [123].

Studies have explored the anti-obesity properties of postbiotics. Balaguer et al. (2022) found that lipoteichoic acid derived from *Bifidobacterium animalis* subsp. *lactis* CECT exhibits anti-obesity effects in a *Caenorhabditis elegans* model by reducing lipid storage [88]. Similarly, Osman et al. (2021) demonstrated that a cell-free extract of *Lactobacillus paracasei* serves as a safer alternative to atorvastatin (ATOR), achieving comparable reductions in serum total lipid, triglyceride, and LDL-C levels in an HFD-induced obesity rat model [124]. Shimizu et al. (2019) investigated the effects of SCFAs on obesity and liver metabolic function [90]. Seven-week-old rats were divided into high-fat diet (control), high-fat diet + 5% SCFA (acetate, propionate, and butyrate), and high-fat diet + 5% cellulose groups. Each diet was fed to the mice for 4 weeks. Although food intake was similar in all groups, body weight and blood glucose levels were significantly lower in the SCFA-fed group than in the cellulose-fed and control groups. Total lipid levels in the liver were also lower, and the expression of genes involved in fatty acid synthesis, such as Fas and Chrebp, was reduced compared with that in the cellulose-fed and control groups [90]. These results suggest that dietary SCFAs may affect fat accumulation and lipid metabolism by directly affecting host signaling. Amiri et al. (2025) conducted an 8-week, randomized clinical trial to evaluate the effects of sodium butyrate (NaBu, 600 mg/day) supplementation in 50 obese adults (BMI 30–40 kg/m^2^) [91]. Participants were divided into either a NaBu or placebo group, both of which followed a hypocaloric diet. Compared with the placebo group, the NaBu group showed a significant reduction in fat mass and visceral fat. In addition, NaBu supplementation significantly upregulated the expression of lipid metabolism-related genes, including adiponectin receptor-1 (ADIPOR1) and uncoupling protein-3 (UCP3) in peripheral blood mononuclear cells (PBMCs), suggesting a role in regulating lipid and energy homeostasis [91].

### 3.5. FMT

Fecal microbiota transplantation (FMT) is a strategy that modulates the gut microbiome by transferring a healthy donor microbiome to an existing microbiome [125]. The goal is to restore the destroyed gut microbial ecosystem [126]. FMT improves the dysbiosis of the gut microbiome by transplanting gut microbes and metabolites, thereby altering the gut microbiome. Therefore, FMT may be an effective therapeutic strategy for obesity treatment. Unlike probiotics, which typically introduce a single microbial strain to influence the gut microbiota, FMT involves the transfer of the entire microbial community, along with its metabolites, providing a broader and more comprehensive approach to resetting the gut microbial balance [127].

Kim et al. (2018) investigated whether FMT from resveratrol-fed donor mice could improve glycemic control and cardiovascular function in obese recipient mice [92]. Resveratrol is known for its anti-obesity properties. In this study, feces from resveratrol-treated mice were transplanted into mice with obesity induced by a high-fat, high-sucrose diet. FMT resulted in improved glucose tolerance without changes in body weight and decreased levels of intestinal pro-inflammatory cytokines such as TNF-α and IL-1β. These findings suggest that FMT is a potential strategy for the treatment of metabolic diseases [92]. Zhu et al. (2024) investigated the effects of FMT in an abdominal obesity (AO) mouse model [93]. AO was induced in mice by a subcutaneous injection of monosodium glutamate (MSG), which led to characteristic symptoms such as gut microbiota dysbiosis and intestinal barrier disruption. FMT from healthy donor mice significantly restored the intestinal mucosal barrier, reduced intestinal permeability and systemic inflammation, and effectively suppressed an increase in abdominal fat and total body weight [93]. However, other studies have reported conflicting results regarding the therapeutic effects of FMT in obesity. A study by Lahtinen et al. (2022) was a randomized, double-blind, placebo-controlled trial to assess whether lean donor FMT could promote weight loss in obese patients [128]. After six months, there were no significant differences between the lean donor FMT group and the autologous placebo group in terms of body composition, metabolic markers (e.g., glucose and lipids), or inflammatory parameters. The mean weight loss was 4.8% in the FMT group and 4.6% in the placebo group, which was not significantly different [128]. Zecheng et al. (2023) conducted a meta-analysis of randomized controlled trials to evaluate the metabolic effects of FMT in patients with obesity and metabolic syndrome [129]. The results showed that FMT significantly improved caloric intake, glucose and insulin metabolism, lipid profiles, and inflammatory markers, whereas its effect on body weight reduction was not statistically significant. These findings suggest that FMT may be more effective in improving obesity-related metabolic parameters than inducing weight loss [129]. Furthermore, He et al. (2022) analyzed the inconsistent outcomes of FMT between patients by investigating the interactions between donor and recipient gut microbiomes [130]. The results showed that recipients with severely disturbed gut microbiota (Enterobacteriaceae-dominant type) generally responded well to FMT. In contrast, recipients with a relatively stable gut microbiota (Bacteroides-dominant type) showed treatment efficacy that varied depending on donor–recipient enterotype compatibility. This study suggests that lower beta diversity between the donor and recipient microbiota increases the likelihood that donor microbes will successfully colonize the recipient gut and exert beneficial effects, highlighting the importance of microbiota compatibility in FMT outcomes [130].

## 4. Perspectives

The emerging evidence presented in this review strongly suggests that the targeted modulation of the gut microbiome is a promising and innovative strategy for addressing obesity. Traditional methods of treating obesity, such as dietary restriction, pharmacotherapy, and bariatric surgery, have limitations in terms of long-term effectiveness and sustainability. In contrast, microbiome-based interventions offer a system-level approach to addressing the underlying metabolic and inflammatory processes that contribute to obesity.

Looking forward, the field must transition from generalized microbiota modulation strategies to precision microbiome engineering. Future research can focus on individualized approaches that consider host genetics, baseline microbiome composition, lifestyle factors, and dietary habits. Metagenomic and metabolomic profiling, combined with machine learning algorithms, can facilitate the identification of microbial signatures predictive of therapeutic responses, paving the way for personalized interventions. Moreover, in evaluation models, the gastrointestinal systems of humans and mice differ in morphological and physiological characteristics, including gut length, transit time, and segment distribution. Given the substantial differences in microbial composition as well, further studies using human in vitro models alongside animal models are needed.

Furthermore, although interventions such as probiotics, prebiotics, synbiotics, and fecal microbiota transplantation (FMT) have shown potential, their long-term safety, strain-specific effects, and consistency across diverse populations require further investigation. The development of next-generation probiotic-engineered microbial consortia and postbiotics may address some of these limitations by providing more targeted and controlled therapeutic options.

Ultimately, integrating gut microbiome modulation into mainstream obesity management will require rigorous clinical validation, regulatory standardization, and public health strategies to ensure accessibility and adherence. As our mechanistic understanding deepens, microbiome engineering could become the cornerstone of precision nutrition and metabolic health.

## Figures and Tables

**Table 1:** Current anti-obesity strategies used by modulating gut microbiome.

Anti-Obesity Strategy	Treatment	Study Model	Diet	Modulation	Anti-Obesity Effects	Ref.
Dietary control	Soluble dietary fiber Fibersol-2	HFD-induced obese mouse	High-fat diet	F/B ratio ↓	Body weight ↓ White fat accumulation ↓	[69]
	Soluble dietary fiber Adzuki bean	HFD-induced obese mouse	High-fat diet	F/B ratio ↓ *Bifidobacterium* ↑	Lipid accumulation, serum lipids ↓ Improve insulin resistance	[70]
	Insoluble dietary fiber extracted from soybean	HFD-induced obese mouse	High-fat diet or HFD + orlistat treatment	*Lactobacillales, Lactobacillus, Lachnospirace* group ↑ *Lachnospiraceae, Bacteroides acidifaciens* ↓	Body weight ↓ TC, TG, LDL-C ↓ HDL-C ↑	[71]
	Insoluble dietary fiber extracted from brown seaweed	HFD-induced obese mouse	High-fat diet	*Akkermansia muciniphila* ↑	Serum cholesterol and glucose ↓	[72]
	Insoluble dietary fiber extracted from pear fruit pomace (*Pyrus ussuriensis* Maxim)	HFD-induced obese mouse	High-fat diet or HFD + orlistat treatment	F/B ratio ↓ *Akkermansia*, *Parabacteroides*, *Alistipes*, *Alloprevotella* ↑	TC, TG, LDL-C ↓ HDL-C ↑	[73]
	Bamboo shoot fiber	HFD-induced obese mouse	High-fat diet or HFD + cellulose treatment	Bacteroidetes ↑ Verrucomicrobia ↓	Body weight, fat mass ↓	[74]
	Herb component berberine	HFD-induced obese mouse	High-fat diet	F/B ratio ↓ SCFA-producing bacteria (*Bacteriodes, Bilophila*) ↑	Body weight, plasma lipid levels, endogenous glucose production, lipolysis ↓ Improve insulin resistance	[75]
	Whole mung bean	HFD-induced obese mouse	High-fat diet	F/B ratio ↓ *Akkermansia*, *Bifidobacterium* ↑	Body weight, fat accumulation, adipocyte size ↓ Improve glucose tolerance	[76]
	*Hirsutella sinensis* mycelium, a medicinal mushroom	HFD-induced obese mouse	High-fat diet	*Parabacteroides goldsteinii* ↑	Body weight ↓	[77]
Probiotics	*Bacteroides acidifaciens*	HFD-induced obese mouse	High-fat diet	-	Body weight ↓ Fat mass ↓ PPARα activation DPP-4 ↓	[44]
	*Lactobacillus paragasseri SBT2055*	HFD-induced obese mouse	High-fat diet	*Bacteroidetes* ↑	Body weight ↓ Fat accumulation ↓ Intestinal fat absorption genes ↓	[78]
	*Lactobacillus gasseri* BNR17	HFD-fed mice	High-fat diet	-	Body weight ↓ Fat accumulation ↓ Leptin ↓	[79]
	*Lactiplantibacillus plantarum GA30*	STZ-induced diabetic mouse		Beneficial bacteria ↑	Hyperglycemia↓ Body weight ↓ GABA ↑	[80]
	*Lacticaseibacillus paracasei K56*	HFD-induced obese mouse	High-fat diet	-	Genes related to lipid synthesis (FAS, PPAR-γ, CEBP-α) ↓ Pro-inflammatory cytokines in the colon (IL-1β, TNF-α, IL-6) ↓	[81]
	*Hafnia alvei HA4597*	Overweight human (BMI 25–29.9 kg/m^2^)	Hypocaloric diet	-	Body weight ↓ BMI ↓ Hip circumference ↓ Satiety hormone ↑	[82]
Prebiotics	Inulin	HFD-induced obese mouse	High-fat diet	F/B ratio ↓	Body weight ↓ Adiposity ↓ Improve glucose tolerance	[83]
	Galactooligosaccharides (GOSs)	HFD-induced obese mouse	High-fat diet	F/B ratio ↓	Body weight ↓ Adiposity ↓ Improve glucose tolerance	[84]
	Fructooligosaccharides (FOSs)	HFD-induced obese mouse	High-fat diet	*Akkermansia, Odoribacter, Roseburia*, and *Muribaculaceae* ↑	Body weight ↓ Fat accumulation ↓ Improve glucose tolerance	[85]
Synbiotics and postbiotics	Synbiotic capsule, containing a combination of *Lactobacillus* spp., *Streptococcus thermophilus*, and *Bifidobacterium* spp. with FOSs	Overweight and obese children	Ad libitum diet	fecal LAB counts ↑	BMI ↓ Serum triglyceride levels ↓ Total and LDL cholesterol levels ↓	[86]
	*Lactiplantibacillus pentosus* GSSK2 + isomalto-oligosaccharides	HFD-induced obese mouse	High-fat diet	F/B ratio, ↓ *Enterobacteriaceae* ↓ *Lactobacillus* spp., *Akkermansia* spp., *Faecalibacterium* spp., *Roseburia* spp. ↑	Body weight ↓ Abdominal circumference ↓ BMI ↓ Visceral fat deposition ↓	[87]
	Lipoteichoic acid derived from *Bifidobacterium animalis* subsp. *lactis* BPL1	*Caenorhabditis elegans* N2	Treatment with live probiotics or lipoteichoic acid extracts or heat-killed probiotics	-	Lipid storage ↓ TG levels ↓	[88]
	Cell-free extract of *Lactobacillus paracasei*	HFD-induced obese rat	High-fat diet or HFD + ATOR	-	Total and LDL cholesterol levels ↓ Body weight ↓	[89]
	SCFAs (acetate, propionate, and butyrate)	HFD-induced obese rat	High-fat diet	-	Body weight ↓ Blood sugar ↓ Fatty acid synthesis genes (Fas, Chrebp) ↓	[90]
	Sodium butyrate	Obese adults (BMI 30–40 kg/m^2^)	Hypocaloric diet	-	Fat mass and visceral fat ↓ Lipid metabolism-related genes (ADIPOR1, UCP3) ↑	[91]
FMT	FMT + resveratrol	Male C57BL/6N mice	High-fat, high-sugar diet	-	Improve glucose homeostasis Intestinal inflammatory cytokines (TNF-α, IL-1 β) ↓	[92]
	FMT	Abdominal obesity (AO) mouse	Normal chow diet	Restoration of gut microbiota	Restore intestinal mucosal barrier Systemic inflammation ↓ Abdominal fat accumulation ↓ Body weight ↓	[93]
	FMT	Adolescents with obesity	Usual diet	Microbial diversity ↑	Improve glucose tolerance Improve insulin sensitivity Abdominal fat ↓	[94]

↑ indicates an increase; ↓ indicates a decrease.

## Data Availability

Not applicable.
